# Supplementary material for: A deficiency screen identifies genomic regions critical for sperm head-tail connection
Source: bioRxiv. 2024 Aug 21:2024.08.20.608819. Preprint. [Version 1] doi: 10.1101/2024.08.20.608819 (PMC11452195; doi:10.1101/2024.08.20.608819)
Supplement: Supplement 1 [file NIHPP2024.08.20.608819v1-supplement-1.pdf]

bioRxiv preprint doi: https://doi.org/10.1101/2024.08.20.608819; this version posted August 21, 2024. The copyright holder for this preprint (which was not certified by peer review) is the author/funder. This article is a US Government work. It is not subject to copyright under 17 USC 105 and is also made available for use under a CC0 license.

Supplemental Material

Supplemental Figures

Figure S1. PLP overexpression does not affect male fertility.

Figure S2. Analysis of screen hit Df(2L)ED1473.

Figure S3. Analysis of screen hit Df(3R)ED10639.

Figure S4. Analysis of screen hit Df(3R)ED5577.

Figure S5. Analysis of screen hit Df(3R)ED4421.

Supplemental Files

File 1

- Sheet 1. List of Dfs tested in this screen. Numbers in the first column correspond to the X-axes of the plots in Figure 2.
- Sheet 2. Results of primary and secondary fertility screens.
- Sheet 3. Statistical information for Figure 3.
- Sheet 4. Statistical information for Figure 4.
- Sheet 5. Statistical information for Figure 5B.
- Sheet 6. Statistical information for Figure S1.

File 2

- Computational methods used in this study.

bioRxiv preprint doi: https://doi.org/10.1101/2024.08.20.608819; this version posted August 21, 2024. The copyright holder for this preprint (which was not certified by peer review) is the author/funder. This article is a US Government work. It is not subject to copyright under 17 USC 105 and is also made available for use under a CC0 license.

Figure S1. PLP overexpression does not affect male fertility

Male fertility is normal in flies overexpressing PLP::GFP using the ubi promoter from a single transgene (n=98) compared to control (n=102). This is the "sensitized" strain background used in the Df kit screen. Some of the control data has also been used in other figures. Each data point is color coded to a given trial. Statistical comparisons done via t-test with Welch's correction. ns = not significant

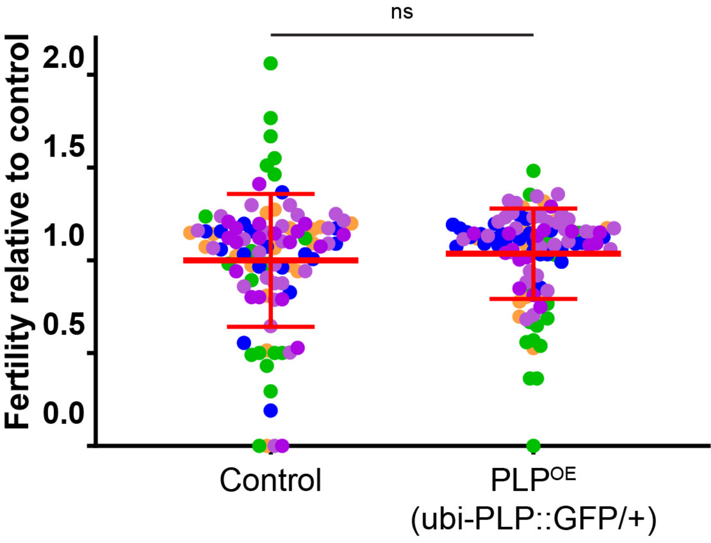

Galletta et al., Figure S1

bioRxiv preprint doi: https://doi.org/10.1101/2024.08.20.608819; this version posted August 21, 2024. The copyright holder for this preprint (which was not certified by peer review) is the author/funder. This article is a US Government work. It is not subject to copyright under 17 USC 105 and is also made available for use under a CC0 license.

Figure S2. Analysis of screen hit Df(2L)ED1473

A. Genomic region of DF(3R)ED1473. Horizontal bars represent regions of the genome removed by Df screen hits and each smaller Df tested. Interaction status color code in inset. Genes removed by the Df from the screen are represented by vertical bars with their width indicating the region of the genome they occupy. Gene color indicates expression level (FPKM) in testis from FlyAtlas 2 (legend in B). Axis is FlyBase Gene Coordinates. Grey region represents the region of the genome discussed in the Results/Discussion. **B.** Heatmap of the FPKM from RNAseq (FlyAtlas 2) of genes in grey region in A. for various tissues. Rightmost column indicates presence or absence of proteins encoded by these genes in the sperm proteome 3. **C.** Heatmap of snRNAseq data from FlyCellAtlas of gens in C. with moderate or higher expression in the testis.

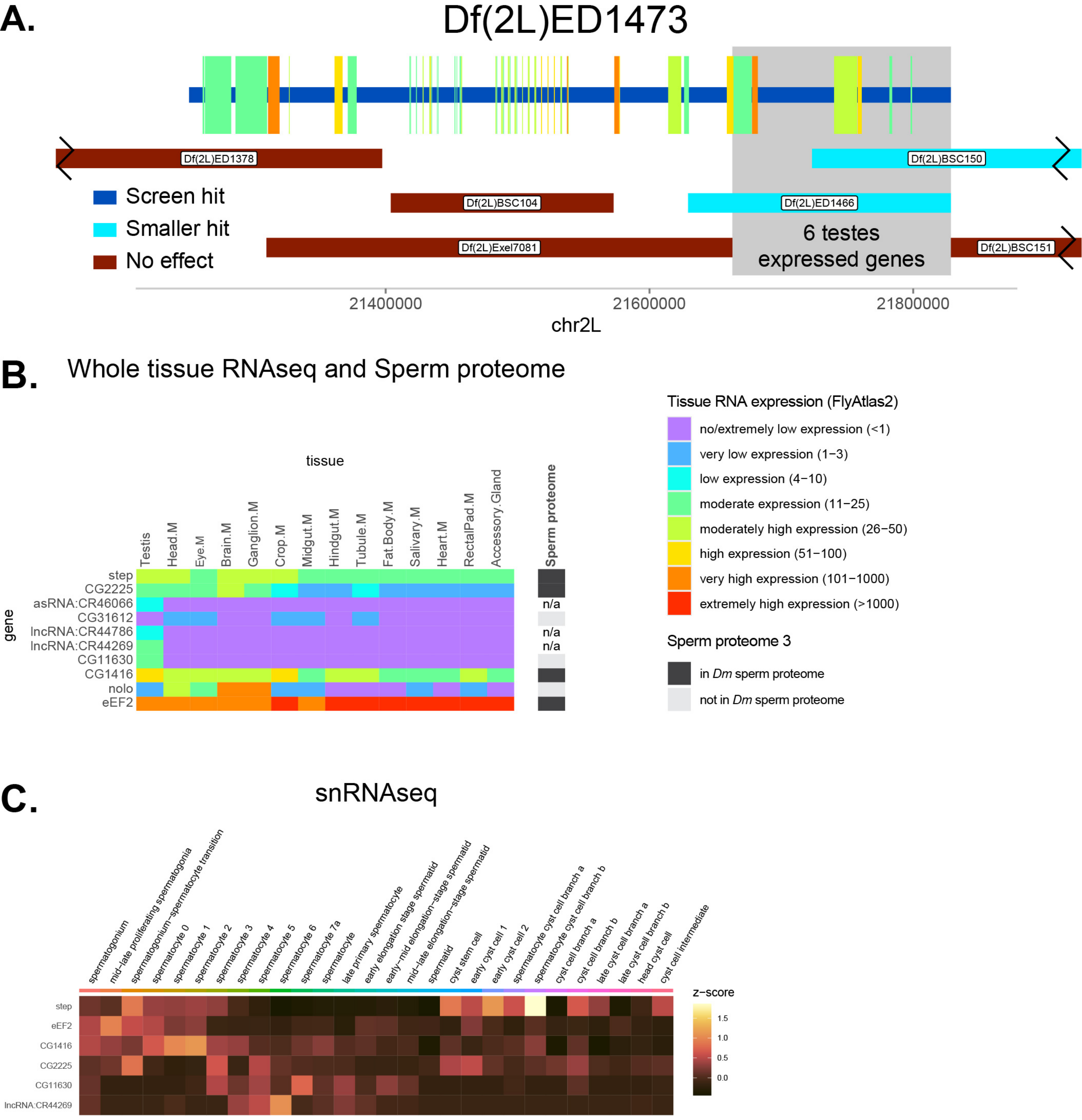

Galletta et al, Figure S2

bioRxiv preprint doi: https://doi.org/10.1101/2024.08.20.608819; this version posted August 21, 2024. The copyright holder for this preprint (which was not certified by peer review) is the author/funder. This article is a US Government work. It is not subject to copyright under 17 USC 105 and is also made available for use under a CC0 license.

Figure S3. Analysis of screen hit Df(3R)ED10639

A. genomic region of DF(3R)ED10639. Horizontal bars represent regions of the genome removed by Df screen hits and each smaller Df tested. Interaction status color code in insets. Genes removed by the Df from the screen are represented by vertical bars with their width indicating the region of the genome they occupy. Gene color indicates expression level (FPKM) in testis from FlyAtlas 2 (legend in B). Except for *asun* only genes with moderate or higher expression are displayed. Axis is FlyBase Gene Coordinates. Regions of the genome discussed in the Results/Discussion are indicated. **C.** Heatmap of the FPKM from RNAseq (FlyAtlas 2) of genes in grey region in A. for various tissues. Rightmost column contains indicates presence or absence of proteins encoded by these genes in the sperm proteome 3. **C.** Heatmap of snRNAseq data from FlyCellAtlas of gens in C. with moderate or higher expression in the testis.

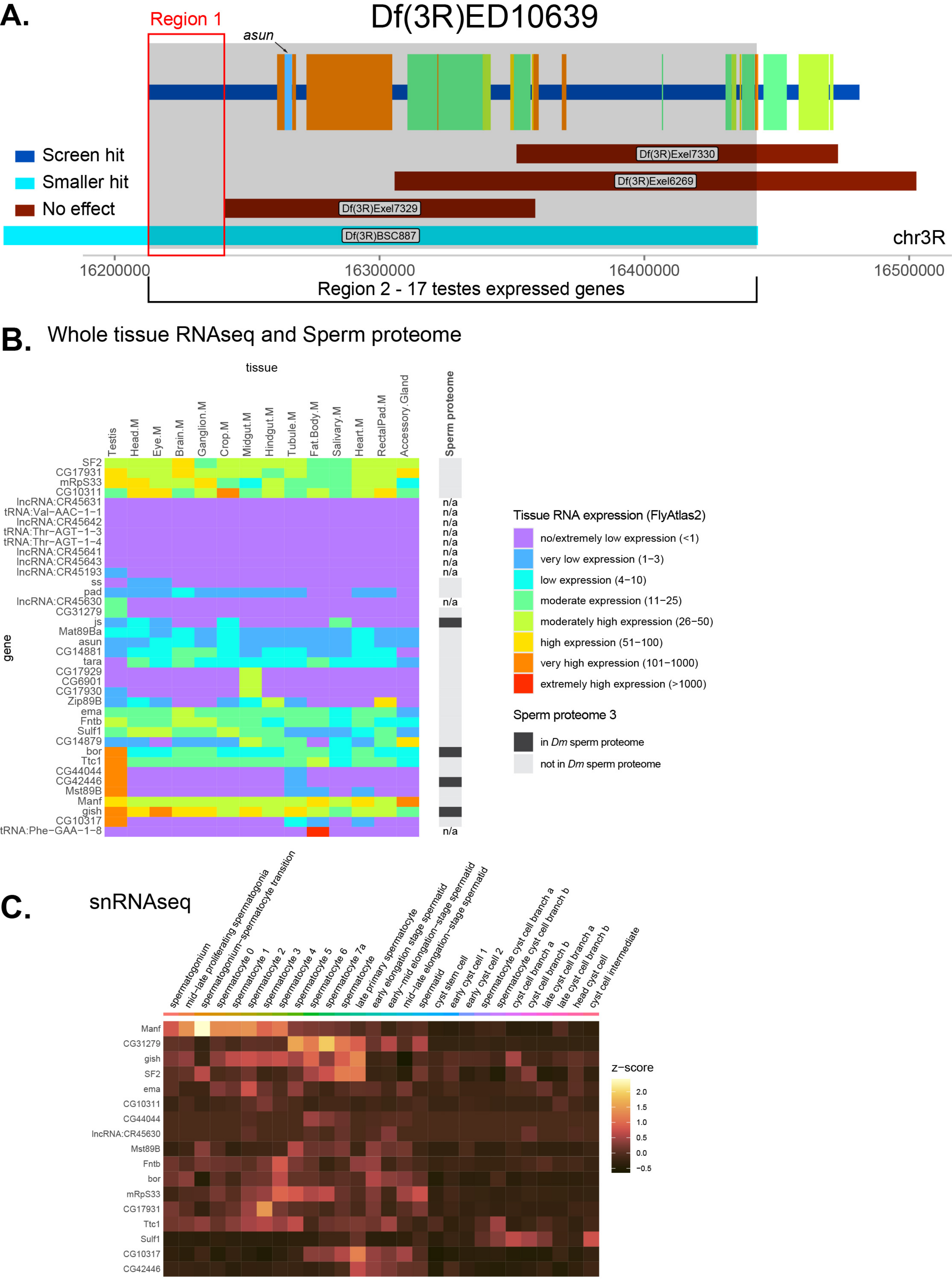

Galletta et al, Figure S3

bioRxiv preprint doi: https://doi.org/10.1101/2024.08.20.608819; this version posted August 21, 2024. The copyright holder for this preprint (which was not certified by peer review) is the author/funder. This article is a US Government work. It is not subject to copyright under 17 USC 105 and is also made available for use under a CC0 license.

Figure S4. Analysis of screen hit Df(3R)ED5577

A. genomic region of DF(3R)ED5577. Horizontal bars represent regions of the genome removed by Df screen hits and each smaller Df tested. Interaction status color code in insets. Genes removed by the Df from the screen are represented by vertical bars with their width indicating the region of the genome they occupy. Gene color indicates expression level (FPKM) in testis from FlyAtlas 2 (legend in B). Axis is FlyBase Gene Coordinates. Grey regions represent the region of the genome discussed in the Results/Discussion. **C.** Heatmap of the FPKM from RNAseq (FlyAtlas 2) of genes in grey region in A. for various tissues. Rightmost column indicates presence or absence of proteins encoded by these genes in the sperm proteome 3. **C.** Heatmap of snRNAseq data from FlyCellAtlas of gens in C. with moderate or higher expression in the testis.

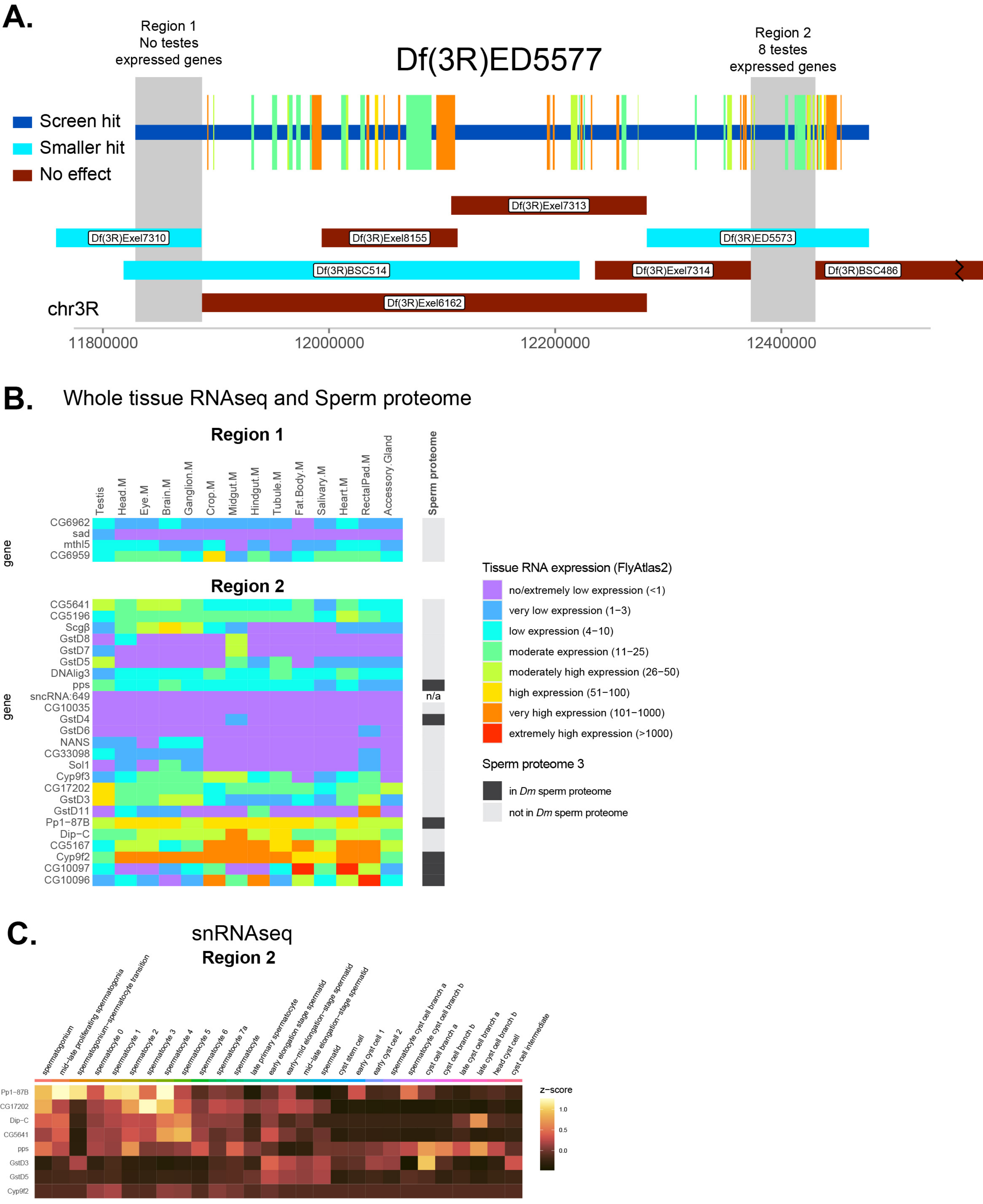

Galletta et al, Figure S4

bioRxiv preprint doi: https://doi.org/10.1101/2024.08.20.608819; this version posted August 21, 2024. The copyright holder for this preprint (which was not certified by peer review) is the author/funder. This article is a US Government work. It is not subject to copyright under 17 USC 105 and is also made available for use under a CC0 license.

Figure S5. Analysis of screen hit Df(3R)ED4421

A. genomic region of DF(3R)ED4421. Horizontal bars represent regions of the genome removed by Df screen hits and each smaller Df tested. Interaction status color code in insets. Genes removed by the Df from the screen are represented by vertical bars with their width indicating the region of the genome they occupy. Gene color indicates expression level (FPKM) in testis from FlyAtlas 2 (legend in B). Axis is FlyBase Gene Coordinates. Grey regions represent the region of the genome discussed in the Results/Discussion and the specific genes discussed are indicated. **B.** Heatmap of the FPKM from RNAseq (FlyAtlas 2) of genes in grey region in A. for various tissues. Rightmost column contains results of presence or absence of proteins encoded by these genes in the sperm proteome 3. **C.** Heatmap of snRNAseq data from FlyCellAtlas of gens in C. with moderate or higher expression in the testis.

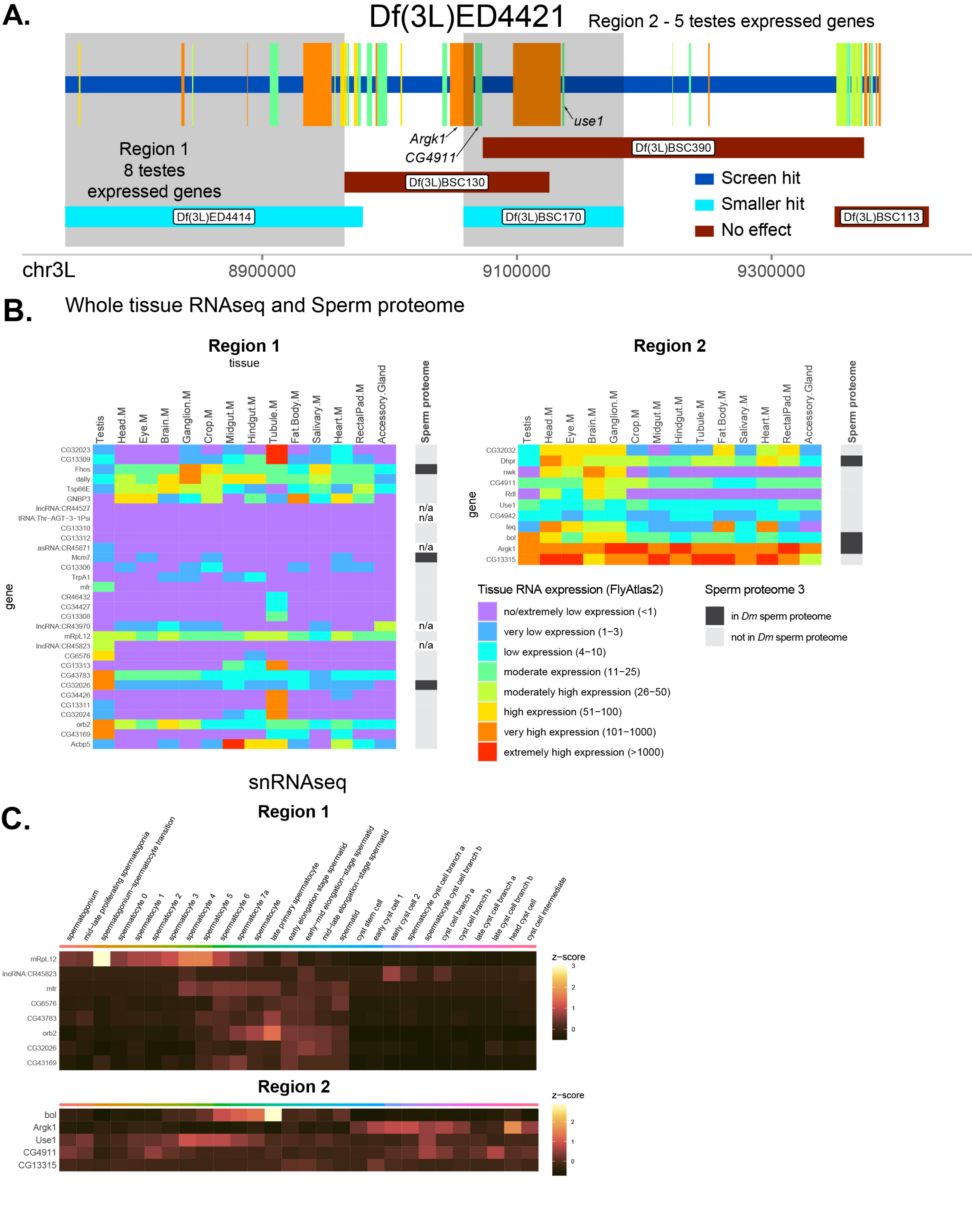

Galletta et al, Figure S5
